# Outlining Potential Biomarkers of Exposure and Effect to Critical Minerals: Nutritionally Essential Trace Elements and the Rare Earth Elements

**DOI:** 10.3390/toxics11020188

**Published:** 2023-02-17

**Authors:** Jill A. Jenkins, MaryLynn Musgrove, Sarah Jane O. White

**Affiliations:** 1 Wetland and Aquatic Research Center, U.S. Geological Survey, 700 Cajundome Boulevard, Lafayette, LA 70506, USA; 2 Oklahoma-Texas Water Science Center, U.S. Geological Survey, 1505 Ferguson Lane, Austin, TX 78754, USA; 3 Geology, Energy & Minerals Science Center, U.S. Geological Survey, 12201 Sunrise Valley Dr., Reston, VA 20192, USA

**Keywords:** adverse outcome pathway, aquatic animals, essential metals, cobalt, chromium, manganese, nickel, zinc, ecotoxicology, lanthanides

## Abstract

Emerging and low-carbon technologies and innovations are driving a need for domestic sources, sustainable use, and availability of critical minerals (CMs)—those vital to the national and economic security of the United States. Understanding the known and potential health effects of exposures to such mineral commodities can inform prudent and environmentally responsible handling and harvesting. We review the occurrence, use, predominant exposure pathways, and adverse outcome pathways (AOP) for human and fish receptors of those CMs that are nutritionally essential trace metals (specifically, cobalt, chromium, manganese, nickel, and zinc), as well as the rare earth elements. Biological responses to some elements having comparable biogeochemistry can sometimes be similar. Candidate quantifiable biomarkers for assessing potential AOP are conveyed.

## 1. Introduction

The fast pace of innovation, population growth, and economic development are driving demands for mineral commodities used in emerging and low-carbon technologies [1]. These include renewable energy technologies, electric vehicles, military applications, wood preservatives, pesticides, and batteries. As defined in the United States (U.S.) Presidential Executive Order 13,817 (2017) [2], critical minerals (CMs) (i) are non-fuel minerals or mineral materials vital to economic and national security, (ii) have supply chains that are vulnerable to disruption, and (iii) serve necessary functions in product manufacturing [3,4,5]. Based on an extensive multi-agency assessment, the U.S. Geological Survey (USGS) has recently delineated 50 CMs [6]. Ensuring their secure and reliable supply necessitates understanding their potential health effects and biogeochemical behavior to inform environmental risks associated with their widespread use and increasing availability.

**Figure 1:**
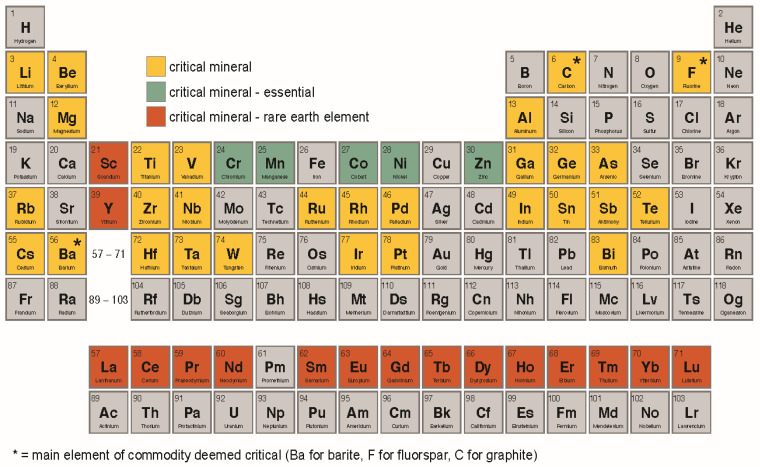
Periodic table highlighting critical elements and ore components defined as critical minerals (CM) in 2022 [6]. Of the CM (highlighted in yellow, green, or orange), the nutrients currently considered essential for humans are green and the rare earth elements are orange [11,16,17,18,19,20].

The CMs (Table 1; Figure 1) derive from diverse geochemical categories. The 17 rare earth elements (REEs) are soft heavy metals with similar properties [7,8]. Other CMs are spread throughout the periodic table (Figure 1), typically situated as alkaline or alkali earth metals, transition metals, and metalloids [9]. Trace elements (or trace metals) occur in low concentrations in rocks, soils, waters, atmosphere, and biota. They can be present in living tissues in small amounts, and some are nutritionally essential for human health [10]. For the known essential elements, essentiality and toxicity are unrelated and toxicity is a matter of dose or exposure; that is, an element can be essential and toxic, depending on conditions and concentration [11]. Other CMs are toxic. For example, antimony (Sb), arsenic (As), beryllium (Be), chromium (Cr), nickel (Ni), and zinc (Zn) are listed on the U.S Environmental Protection Agency (EPA) Priority Pollutant List, a set of regulated chemical pollutants for which the EPA has published analytical test methods [12]. Additionally, some CMs are included in the Report on Carcinogens [13], a scientific and public health document that identifies and discusses agents, substances, or exposure circumstances that may pose a cancer hazard to humans (Appendix A Table A1). For many CMs, toxicity is poorly understood, though ~80% are included on the U.S. Agency for Toxic Substances and Disease Registry (ATSDR) Priority List of Hazardous Substances (Substance Priorities List) [14] (Table A2). This list seeks to prioritize substances of greatest human health concern, due to known or suspected toxicity and the potential for exposure at facilities on the National Priorities List [https://www.epa.gov/superfund/superfund-national-priorities-list-npl, accessed on 9 August 2022]; these substances are or should be considered as candidates for toxicological profiles [14].

Increasing demand for CMs imparts pressure on natural resources [1,15]. The economic valuation of these elements is often reflected by low abundance and difficult accessibility [4]. The mining and extraction of many metals is energy-intensive and can lead to elevated concentrations of metals or associated byproducts in surrounding soil and water. Primary metal production, industrial processing, and the widespread use of CMs in products and industries have resulted in numerous releases to the environment, often via outflow of effluent from mines, landfills, factories, and metal refineries [15].

We review adverse outcome pathways (AOP) that are known or likely to occur for humans and fish to understand the potential health effects of the increasing production, use, and environmental distribution of CMs. The AOP approach is useful for delineating mechanisms and potential health effects and facilitates understanding data from environmental monitoring of concentrations [21]. A suite of biomarkers can include biotransformation enzymes, oxidative stress-, reproductive-, endocrine-, genotoxic -, and physiological and immunological parameters [21]. Because fish cannot escape the water in which they live, they have been studied to reflect pollution levels and biological effects [22,23,24,25,26] and are considered organisms of choice for monitoring and assessing the effects of pollution in aquatic ecosystems [27]. Reliable biomarkers along an AOP are sensitive, quantifiable indices of both pollutant bioavailability and early biological responses, often elucidating cause-effect and dose-effect relationships in health risk assessments [21]. Biomarkers are indicator signals in biological systems that are indicative of effects or exposure to an environmental agent, which may be chemical, physical, or biological [28]. A biomarker of exposure is the identification of an extraneous substance within the system, or the interactive product between a contaminant and a biological structure [21]. For example, plasma from all smallmouth bass *Micropterus dolomieu* sampled from the U.S. Mid-Atlantic region Chesapeake Bay contained perfluoroalkyl substances (PFAS), indicating exposures to PFAS [29]. Biomarkers of effect are measurable changes in an individual that indicate health impairment or disease, whereby biological structures, functional capacity, or systems are altered [21,30]. For example, reductions in numbers of a dendritic-like cell in flame retardant-exposed American kestrels (*Falco sparverius*) affect their innate immune response [31]. Such an understanding of events along an AOP following exposures is foundational to determining CM-associated risks, and can provide insight for avenues for research into those risks.

To proactively assess potential health risks for increased physical contact with CMs for humans and fish, we review the occurrence, use, exposure, and toxicological mechanisms associated with CMs that are also considered essential elements (specifically, cobalt (Co), Cr, manganese (Mn), Ni, and Zn), along with the REEs. While numerous studies of essential elements and their health effects are available, the REEs are less studied, and thus are helpful to include for comparison. We consider REEs as a group, rather than individually, both because of a paucity of related studies and because they have similar chemical properties.

## 2. Aquatic Studies and Environmental Parameter Considerations

Aquatic organisms take up and accumulate trace metals, whether essential or not [32]. Metals dissolved in water may be accumulated by direct adsorption to body surfaces with subsequent uptake across permeable membranes, such as gills [33], with particulate metals being accumulated by ingestion and through food web trophic transfer [32]. Several metals target the active ionic uptake pathways on gills, likely using the pathways as a route of entry through “ionic mimicry” [19]. For example, Co, Zn, cadmium (Cd), lead (Pb), and strontium mimic calcium (Ca), Cr and molybdenum mimic sulfate, and Ni mimics magnesium (Mg), whereby these acts of mimicry may reduce uptake of the essential element at the gills by competition [19]. Regardless, acute exposures target gills, which generally comprise over 50% of the surface area of the fish and are in intimate and continuous contact with the external water [19]. Morphological changes in gills cause a generalized inflammatory response, resulting in rapid death by suffocation due to edematous swelling, cellular lifting and necrosis, lamellar fusion, and impeded blood and water flow through and across the respiratory lamellae [19]. When comparing fish to human exposures, the drinking water thresholds designed to protect human health should not be confused with water quality criteria used to guide environmental protection; the human digestive tract is far more resistant to most metals than the gills of fish or aquatic invertebrates [19].

In the water phase, environmental factors such as water temperature, dissolved oxygen concentration, pH, hardness (i.e., dissolved Ca and Mg), salinity, alkalinity, and dissolved organic carbon influence metal bioavailability, accumulation, and toxicity [22], and are key to understanding CM behavior in aquatic ecosystems. Metals are partitioned in dissolved form, adsorbed to suspended matter, or can preferentially accumulate in sediments and be retransferred to the water column and enter the food chain [34]. Ionic forms or simple inorganic compounds can be, but are not always, more toxic than complex inorganic or organic compounds [19]. Often (but not always) in fresh water, lower pH increases the free ion concentration, thereby increasing toxicity, whereas alkalinity (e.g., from dissolved carbonates and other ions) and inorganic anions tend to complex metal ions, thereby decreasing toxicity [19]. Organism uptake and assimilation responses vary among trophic levels and taxa, even for species that are biologically similar, complicating the understanding of metal cycling in food webs and organism responses [35]. Comparative studies of different aquatic organisms indicate that some species or groups likely have variable resiliencies to trace metal contamination [32]. Moreover, aquatic species can be genetically adapted to local levels of metals, so responses of fish species in different regions may not always be directly comparable [19]. The accumulation and distribution patterns of toxic metals in fish tissues also depend on rates of uptake and elimination [36].

We report benchmark concentrations where they exist for the CMs discussed to provide context, considering several sources:

- EPA national primary drinking water standards (maximum contaminant levels or MCLs), which are regulatory benchmarks for drinking water for protecting human health [37];
- EPA secondary drinking water standards, which are non-regulatory aesthetic benchmarks for drinking water [38];
- Non-regulatory health-based screening levels (HBSLs), developed by the USGS for contaminants without EPA standards or guidelines to place water quality data in a human health context [39];
- In the absence of any of the above, World Health Organization drinking water quality guidelines [40]; and
- Sediment quality guidelines (SQGs) for freshwater ecosystems [41] to provide environmental context for aquatic species; specifically, the Probable Effect Concentration [PEC], which is the concentration above which bed sediments are shown statistically to frequently have adverse effects on benthic biota.

## 3. Essential Trace Elements

Metal ions are unique among nutrients because they cannot be synthesized or degraded by metabolism [19]. Many animal enzymes require metals for catalytic activity [15,16], yet even essential metals can be toxic and pose serious health risks if not processed in the body properly, or if they bio-accumulate at sufficiently high concentrations [15,17,18]. For animal physiology and toxicology, metal speciation is highly important, influencing cellular interactions and entry into cells. The simplest form of metal speciation is whether it is dissolved or in particulate form [19,42].

Trace elements are present in the body in small amounts or at low concentrations. Some are nutritionally essential for life; others, such as vanadium (V) and boron (B), might be [11,17,18,19,20]. Essential elements for aquatic organisms are still under discussion [43], thus for the sake of this document, those of likely essentiality for both fish and humans are the focus. The major determinant of metal ions being essential for life is the requirement by a substantial fraction of enzymes for trace metal involvement in enzyme catalytic activity, as well as their contribution to metabolic activity. Trace elements may also facilitate the generation of end-products from substrates [16,44]. The most common essential metal is iron (Fe), often participating in energy metabolism and playing a vital role in oxygen transport as a constituent in hemoglobin and myoglobin [16,18]. Of the CMs, essential micronutrients include Co, Cr, Mn, Ni, and Zn [19], with typical nutritional thresholds of <1–100 mg/day, versus macronutrients (e.g., Ca, Mg, and potassium) at >100 to thousands of mg/day [42].

Biomarkers of exposure include metals in the biological matrices, such as toenails, bone, hair, urine, blood, exhaled breath condensate, fish otoliths [ear bones], and scales [17,45,46,47,48,49,50,51,52,53], with REE signatures being detected in penguin feathers [54]. Molecular initiating events occur when a compound and biomolecule or biosystem interact. For the essential nutrients, the initiating events that can be linked to an AOP can be considered potentially conserved across animal taxa [55].

### 3.1. Cobalt

Cobalt is a transition metal, similar in properties to adjacent metals Fe and Ni (Figure 1). It occurs in the +2 and +3 oxidation states, can substitute for Fe in ferromagnesian rocks and minerals, and occurs commonly with copper (Cu), Fe, Pb, Ni, and silver (Ag) ores [56,57]. Cobalt is widely dispersed in the environment, though concentrations are usually low; it is often combined in the environment with oxygen, sulfur, and As [58]. Concentrations are relatively high in ultramafic and mafic igneous rocks. The ionic radius of Co is similar to that of Mg, Mn, Fe, and Ni, for which Co can substitute in many minerals. It is cycled through natural processes like volcanic eruptions and weathering, and can be introduced through anthropogenic activities such as burning coal or oil, or the production of Co alloys [59]. Co(II) is more stable than Co(III) in aqueous solution and is present in the environment and in commercially available Co compounds [60]. Concentrations in natural waters are strongly influenced by coprecipitation or adsorption by Fe and Mn oxides [61]. Cobalt has a health-based screening level (HBSL) of 2 μg/L [39].

Cobalt has been used as a blue pigment for thousands of years and in industrial applications, beginning in the 1900s [56]. In the United States, Co and its other chemical forms are generally obtained from imported materials and by recycling scrap metal that contains Co, rather than by mining [58]. Most Co is produced as a by-product of Cu and Ni production [13,56,57]. Cobalt is used primarily in rechargeable batteries, solar and power renewable energies, high-strength metal alloys, and data storage applications [57]. Two short-lived radioactive isotopes of Co associated with nuclear reactors are used in medicinal applications, as are Co nanoparticles [13].

For most people, consumption of food and drinking water are the primary exposure pathways for Co, and it can be concentrated in meat and dairy products [14]. Cobalt, being the central atom in vitamin B12, is vital for its structure and function, and is required for proper cellular growth and function, red blood cell regeneration, energy production, and metabolism [42,56,57,60,62]. The recommended dietary allowance of vitamin B12 is 2.4 μg/day, containing 0.1 μg of Co [14]. Excessive Co exposure by dietary supplements, and in occupational, environmental, and medical settings can result in neurological (e.g., visual and hearing impairment), cardiovascular, and endocrine deficits [63]. Industrial inhalation, waste management, and mining activities are important exposure pathways [42,57,63,64]. Primary target organs for Co are the skin and respiratory system [63].

Key events related to Co toxicity and carcinogenicity in humans depend on the cellular uptake and intracellular release of Co ions from particles *in vivo*. Cobalt and Co compounds that release Co ions in vivo are anticipated to be human carcinogens [13] (Table A1 and Table A2). Water-soluble ions enter cells through ion channels within cell membranes, and poorly soluble particulate Co compounds are taken up by lysosomes via endocytosis; Co is then solubilized in the lysosomal acidic environment and the ions are released [13]. This process, in essence, makes the Co ions truly “bioavailable” intracellularly. The redox active Co ion catalyzes the generation of reactive oxygen species (ROS), with toxicity mainly arising from its competition with other biologically essential metal ions, thus inhibiting proper function of macromolecules [64]. Oxidative stress results from the point at which the production of ROS exceeds the capacity of intracellular antioxidants to prevent damage [65].

At the cell surface, the critical first points of contact are lipids and biomembranes, with influences on bilipid membrane morphology and asymmetry [42]. Biological competition between Fe and Co is acute, as these metal ions have similar ionic radii and the same biologically relevant oxidation states (2+/3+) [64,66]. At the plasma membrane, metal-specific protein ion channels are needed for influx and efflux of Co and Mn (and Ni), including divalent metal transporter 1 (DMT1) and dopamine transporter (DAT). Transferrin (the glycoprotein responsible for Fe transport) receptors, and Zn transporters (ZIP8 and ZIP14), also are responsible for influx of other divalent cations and ligands, not being specific to the metals [42]. When cytosolic concentrations of a metal reach a threshold, efflux mechanisms are upregulated to excrete the excess, such as lysosomes and secretory vesicles loaded with ions that fuse with the plasma membrane for release into the extracellular matrix. The uptake and regulation of Co involves a cascade of metellochaperones and metal-ion storage proteins, such as metallothioneins (MT) (highly conserved, cysteine-rich metal-binding proteins important for homeostasis and buffering against heavy metals) [42]. Both Co and Ni affect ATP production, altering mitochondrial permeability transition pores [42] and membrane potential. Because soluble Co is more tolerated than soluble Ni, more mutations by Co can be propagated, supporting findings that Co can be carcinogenic in lung tissue [42]. Metal ions such as Co^2+^ and Ni^2+^ can bind to DNA, generating free radicals, possibly influencing methylation and DNA repair systems [42]. Free radicals are reactive molecular species containing one or more unpaired electrons [67]. Thus, live cell apoptosis assays, mitochondrial membrane potential, and DNA fragmentation or micronuclei presence, as well as receptor and protein presence, and their gene expressions, would be candidate biomarkers of effect.

For fish, foraging is the important pathway to acquire Co and essential metals, with feeding strategies influencing assimilation at the interspecific level [35]. Gills and skin are other routes for uptake of waterborne Co [60,68]. The physiological role of Co for fish is as an intrinsic part of vitamin B12 [60]. Bioaccumulation of Co is similar to that of other divalent metals, including Cu, Cr, Zn, and Pb, which bioaccumulate in gonads [46].

While the specific transport systems aforementioned likely occur in fish, the molecular mechanisms of Co toxicity and subsequent physiological effects in fish are understudied [60]. Possible processes are inferred from mammalian studies, with almost all information on the biochemistry of Co derived from them [46,60]. As stated, main routes of uptake are via fish gills and gut, whereby increasing Ca levels decreases Co uptake [60]. Calcium channels provide a gateway for the uptake, and cell membrane Ca^2+^-ATPases are important for efflux [60]. In one study with male zebrafish (*Danio rerio*) chronically exposed to Co for 12 days, fertilization and hatching were reduced, and a concentration-dependent increase in DNA strand breaks was detected in sperm; DNA damage returned to control levels after recovery for 6 days, and DNA repair genes were upregulated in testes [46]. The interplay of dietary and waterborne metal uptake has yet to be explicitly demonstrated, with fish exploratory behavior reduced, growth lessened, and feed intake lowered with both Co and Ni exposures [68]. Cobalt has been used to block the lateral line of the fish, potentially influencing responses to prey [60]. With the paucity of studies with Co in fish, future investigations could examine chronic toxicity under environmentally realistic scenarios, identify transporters involved in Co uptake, and characterize the molecular mechanisms of toxicity and resulting physiological effects [60].

### 3.2. Chromium

Chromium occurs throughout the environment in air, water, and soil, in several oxidation states [69]; more stable oxidation states are trivalent Cr (Cr(III)) and hexavalent Cr (Cr(VI)) [13,70]. The oxidative states differ in their mobility, bioavailability, and toxicity, with Cr(III) considered to be essential nutritionally for humans and fish, whereas anthropogenic Cr(VI) is toxic and a known human carcinogen (Table A1 and Table A2) [13,19,71]. Cr(VI) compounds are strong oxidizing agents, corrosive, and are generally reduced to Cr(III) [13,72]. Cr(VI) forms anionic complexes, primarily chromate (CrO_4_^2−^) and dichromate (Cr_2_O_7_^2−^) [26]. Chromium in water originates from weathering of rocks and precipitation, with elevated concentrations attributed to industrial wastewater; Cr in soils originates from fallout due to combustion and geochemical processes such as weathering and volcanic eruptions [71,73]. Chromium is listed on the U.S. EPA’s Priority Pollutant List [12]. The EPA drinking water MCL for total Cr = 0.1 mg/L, which is greater than the WHO guideline of 0.05 mg/L. The PEC of Cr in sediment is 111 mg/kg (dry weight).

The main applications of Cr are in the metallurgical and leather tanning industries, with chromate ore in the United States being used for the production of thousands of tons of products, leading to concern over Cr(VI) pollution [13,26,71]. Cr(VI) compounds function as corrosion inhibitors, in the manufacture of pigments, wood preservatives, and pesticides; these compounds are crucial to the U.S. Department of Defense National Stockpile for use in aircraft fuselages, with the United States being one of the world’s leading producers of Cr compounds [13,71]. Chromium compounds are both imported and exported [74,75].

Dietarily, Cr(III) is needed in low concentrations, for metabolic pathways involving glucose, proteins, and lipids; Cr(III) forms complexes with nucleic acids and proteins [70]. It is involved in oxidation-reduction reactions, insulin regulation [70,71,76], and has been considered a pharmacologic agent [70,75,76]. Drinking water and foods such as grains, meats, and vegetables contain Cr(III) [69,71]. Deficiencies, although not clearly associated with disease, have been reported to lead to altered metabolism. Large doses of Cr(III) supplements may lead to improved glucose metabolism in type 2 diabetes, yet concerns exist about health effects of Cr compounds in them [70,71,75,76].

Although weathering mobilizes Cr, its release into the environment from anthropogenic activities are much greater [77]. The general human population may be exposed to Cr(VI) compounds through inhalation of ambient air, cigarette smoke, ingestion of contaminated water, and dermal contact with products such as pressure-treated wood [13,78]. In human plasma, Cr is bound to proteins, such as transferrin, and is readily transported and diffuses to organs, with most eliminated via kidneys [79]. People and animals near industrial Cr(VI) use or disposal sites have the greatest exposure potential [78]. Hexavalent Cr is highly soluble in water and thus mobile in the aquatic environment, with the stable anionic compounds oxidizing organic matter on contact and yielding oxidized organic matter and Cr(III) [79]. The residence time of Cr in lake water has ranged from 4.8 to 18 years [75]. Chromium is taken up by fish gills and distributed via the blood to a variety of tissues, but the mechanism of uptake across the gills is unknown [79]. Similarly, the toxic mechanism of Cr in fish is unknown, but histopathologies, particularly of the gastrointestinal tract and kidney, show these organs play an important role in Cr toxicity [77].

The different toxicities of Cr(III) and Cr(VI) can be explained by physicochemical properties differences, namely their ability to cross biological membranes [78]. The induction of intracellular damage is determined by differences in size, structures, and charges, with Cr(VI) existing mostly as chromate anions (CrO_4_^2−^) at physiological pH; the anions are able to move easily across cellular membranes using the non-specific phosphate/sulfate anion transporters [72,78]. The larger sized, octahedral structure of Cr(III) ions disallows such easy transport, but a small fraction of Cr(III) salts are taken up into cells through phagocytosis, and poorly water-soluble chromates <5 µm can be phagocytosed and gradually dissolve in the intracellular milieu [78]. Although Cr(III) can bind to DNA, it is non-carcinogenic because of its inability to pass through the membrane transporters [72].

Experimental data for human cancer due to Cr have been inconclusive because of co-occurring carcinogens [13]. Biomarkers of exposure include bodily fluid concentrations and exhaled breath condensate; biomarkers of effects include respiratory and skin manifestations, low birth weights, tumors, and oxidative damage [28]. The mechanisms for cytotoxicity and carcinogenicity by Cr(VI) indicate oxidative deterioration of biological molecules, and decreased levels of protective ascorbic acid and glutathione [72]. Without these adequate intracellular reductants or with overexposures, Cr(VI) compounds induce assaults on DNA such as fragmentation, gene mutations, and sister chromatid exchanges [13,72]. Such alterations are responsible for heritable effects on germ cells, posing risks to future generations [76]. Because Cr is involved in the metabolism of carbohydrates, lipids, and proteins mainly by increasing the efficiency of insulin, abnormal levels influence metabolism [80]. The efficiency of Cr transport into cell membranes depends on insulin concentration, whereby Cr and Fe compete for binding to transferrin, thus illustrating the complexity of metals biochemistry [80].

Toxicity in aquatic ecosystems depends on both biotic as well as abiotic factors, as previously discussed [73,79]. No specific toxic mechanism for acute Cr exposure has been identified in fish, however symptoms were noted in fertilization, hematology, increases in ROS and glycogen levels, tissue histology, and enzyme inhibitions [73,79]. In a study using multiple fish species, Cr occurrence depended on fish age, developmental stage, and species, as well as environmental parameters [73]. Behavioral modifications included suspense of feeding, erratic swimming, mucus discharge, color change, coughing and yawning, and ventilation frequency change that suggests respiratory stress or gill irritation [73,79]. Neither has a single mechanism or impairment been shown with chronic Cr toxicity in fish [79]. Data on the genotoxic potential of Cr(VI) in aquatic animals are scarce, yet one study with goldfish (*Carassius auratus*) using multiple biomarkers at various levels of biological organization demonstrated dose-dependent-DNA damage, with noted effects in the kidneys [26]. In another study using multiple biomarkers along an AOP, Cr(VI) was shown to induce biomarkers of exposure, including expression of metallothionein, tissue lipid peroxidation, and superoxide dismutase activity; biomarkers of effect were demonstrated by gill epithelial hyperplasia and lessened growth with decreased feeding behavior in rainbow trout (*Oncorhynchus mykiss*) [81]. This study concluded that gills were more sensitive to Cr(VI) toxicity, but hepatic tissues played a larger adaptive response to it [81]. No significant bioaccumulation or biomagnification of Cr has been shown to occur at environmentally relevant concentrations [79]. Genotoxicity was demonstrated in Prussian carp *(Carrassius gibelio)*, by Cr(III) and Cr(VI) demonstrated by micronuclei in erythrocytes [82]. With the paucity of thorough information on both acute and chronic Cr exposures to fish, future investigations at the cell to the organismal level, in freshwater, estuarine, and saltwater aquatic ecosystems could be helpful.

### 3.3. Manganese

Manganese is a naturally abundant metallic element in the Earth’s crust, and is ubiquitous in water, soil, air, and food [83,84,85]. While Mn is not a significant constituent of common rocks or minerals, it substitutes for Fe, Mg, and Ca in silicate structures [61]. Manganese is a silver-colored metal that commonly occurs with oxygen, sulfur, and chlorine, with MnO_2_ as the most common form [85,86]. There is only about one-fiftieth as much Mn in the Earth’s crust as there is Fe [61]. Both Mn and Fe have multiple valence states and participate in redox processes in weathering [61]. Manganese does not exist in nature in elemental form but occurs in both organic and inorganic forms; it is an essential nutrient in humans, animals, and plants [83,86,87]. Dietary sources of Mn include nuts, legumes, seafood, and tea [88]. Manganese has a U.S. EPA secondary drinking water standard of 0.05 mg/L for aesthetic concerns, and a HBSL of 0.3 mg/L.

The United States does not produce Mn and is reliant on imports [86]. Manganese is the fourth most industrially used metal, used for steel manufacture, batteries, glass, chemicals, and textiles [84]. Manganese has also been used as a gasoline anti-knock additive [89], and in magnetic resonance imaging [87]. These industries produce extensive wastes containing Mn, despite some active Mn recovery efforts [84,86,87]. Environmental contamination occurs primarily from landfill dumping, mostly from discarded batteries [84], via leaching into waterways. Large-scale surveys in groundwater have reported widespread occurrences of elevated Mn, attributed to land surface-soil-aquifer connections [90].

As an essential element, Mn is required for normal amino acid, lipid, protein, and carbohydrate metabolism [91]. Manganese is a cofactor for many enzymes such as the free radical detoxifying superoxide dismutase (SOD) typically found in mitochondria [85,89]. Inhalation exposure to Mn dusts can lead to lung inflammation. The brain is the major target organ for Mn toxicity, retaining Mn much longer than other tissues [85,92]. Concentrations there can cause Parkinson-like symptoms in adult humans with a movement disorder called manganism [87,89,93]. Manganese affects the central nervous system, with ROS generated neurotoxicity with apoptosis and/or necrosis in neural or glial cells [94]. Occupational exposure risks are high for workers such as miners, welders, steel makers [87] and smelters.

Due to the relationship between Mn’s essentiality and toxicity, Mn homeostasis is vital for organismal function [85]. Toxicity due to overexposure is more prevalent than deficiency [91]. Various protein transporters are involved, including the transmembrane DMT1, representing a large family of metal transporters that are highly conserved from bacteria to humans. DMT1 can transport Zn^2+^, Mn^2+^, Co^2+^, Cd^2+^, Cu^2+^, Ni^2+^, Pb^2+^, and Fe^2+^ [85]. If dietary Fe levels are low, greater absorbance of Mn could occur because of the ions competing for the same transporters [16]. Unlike Fe^2+^, Mn^2+^ does not promote the generation of ROS, and thus is important in diminishing oxidative stress by substituting Fe species [5]. Up to 5% of ingested Mn is absorbed via the gastrointestinal tract, and once absorbed in plasma, it is bound to plasma proteins and transported to the liver [87]. Biliary excretion is the major elimination pathway. Manganese is cholestatic in cattle and rodents [92]. Biomarkers of exposure might be investigated by DMT1 gene expression, Mn efflux transport, as well as inflammatory genes, and cellular responses of apoptosis and mitochondrial dysfunction [28,85,95]. Other biomarkers of exposure can include Mn levels in teeth, hair, and blood, but because it is primarily sequestered in tissue and within intracellular compartments, blood concentration may be a poor biomarker [28,96]. Biomarkers of effect include neurological deficits, altered hormonal, reproductive, cardiovascular, dermatological, hematological, learning and memory, immunological responses, and especially brain basal ganglia histopathology [28,83,87,95].

Some studies on invertebrates and fish have occurred over the last decade [91]. Manganese uptake increases with temperature, decreases with pH, and increases with decreasing salinity [91]. The majority of studies on Mn and aquatic species have focused on hepatic and developmental effects, antioxidant responses in various tissues, and hematologic status [91]. When goldfish *Carassius auratus* were exposed to sublethal doses of Mn, biomarkers of effects included increased SOD, catalase, and glutathione-S-transferase in blood, increased glucose and cortisol, decreased protein, and altered differential cell counts [91]. Moreover, several parameters of the blood profile were changed, and micronuclei indicated genotoxic effects [91]. In commercially fished estuarine species, a radiotracer study with Cd, Mn, and Zn showed clams (*Katelysia scalarina*) were more sensitive than whiting (*Sillago ciliate*) or prawns (*Metapenaeus macleayi*) to the accumulation of these metals, partially explaining the persistence of that fish species in contaminated estuaries [32]. In a study with the freshwater striped gouramie, *Colisa fasciatus*, experimental Mn poisoning resulted in a decreased hepatosomatic index; erythrocyte and leukocyte counts decreased and increased, respectively [97]. Hematological parameters were similarly affected in the freshwater Eastern Asian fish, *Garra gotyla*, indicating disturbance in homeostatic defense abilities [98]. Otolith microchemistry was successfully applied in marine catfish, *Genidens genidens,* as a biomarker of exposure in estuaries polluted by metals from mine tailings [50]. Finally, in short-term tests with cells, carcinogenic activity by Mn compounds were lower than with Cr and Ni; further research in the relation between mutation induction and carcinogenicity is warranted for Mn [89].

### 3.4. Nickel

Nickel is a transition metal that is widely distributed in the environment [99], and is situated near, and closely related to, Fe and Co on the periodic table (Figure 1) [100]. Nickel is more abundant than Co in the Earth’s crust, though both can substitute for Fe in minerals and tend to coprecipitate with Fe and Mn oxides [61]. Natural sources of atmospheric Ni include dusts from volcanic emissions and weathering of rocks and soils [101]. Anthropogenic Ni is released from stacks of furnaces making alloys or from power plants and incinerators; it can take more than a month to settle [102]. It can be released in industrial wastewater, and Ni in soil or sediment attaches to particles containing Fe or Mn [102]. Nickel is ubiquitous in marine and freshwater ecosystems and is well established as an essential nutrient for terrestrial animals and plants; it may also be so for aquatic animals, and it does not appear to concentrate in fish [102,103]. Nickel is listed on the U.S. EPA’s Priority Pollutant List [12]. It has a HBSL of 0.1 mg/L, a WHO guideline of 0.07 mg/L, and a sediment PEC of 48.6 mg/kg (dry weight).

Nickel is used in numerous industrial practices for its anticorrosive properties, especially in the production of stainless steel and corrosion-resistant alloys for commodities such as coins, jewelry, machinery, and household utensils [13,55,99,100]. Nickel compounds are used for electroplating, color ceramics, and battery production [102]. Exposure to the general population is by inhalation from wind-blown dust derived from natural and anthropogenic sources, especially from tobacco smoke [104]. Exposure is also via oral and dermal routes; the most commonly reported adverse health effect is contact dermatitis, especially from exposures to jewelry [102]. Occupationally exposed workers suffer respiratory distress which may be compounded by other co-occurring elements such as Cr [102]. Food is a major source of Ni exposure, although exposure and sensitivity can also occur from body piercings and artificial body parts [102]. Biomarkers of exposure are levels of Ni in blood, urine, and body tissues, as well as tumorous growths and epithelial dysplasia [99].

Epidemiological studies have demonstrated that Ni compounds are human carcinogens, and some are carcinogenic in animal bioassays [13,105]. Lung, nasal, and pharyngeal cancers have been documented in Ni refinery workers, with cellular micronuclei and histone modifications as biomarkers of effect [101,105]. A positive dose response was noted between lung cancer occurrence and water-soluble Ni, with a long latent period prior to cancer diagnosis (10–40 years) and with other tumors presenting in the interim [105]. Renal effects were noted after inhalation and urine excretion of Ni, and a number of immunological and lymphoreticular effects have been noted in humans and animals [102]. After inhalation, alveolar macrophage hyperplasia was found, and some studies have shown Ni compounds can induce atrophy of nasal olfactory epithelium [102]. A relationship between Ni and Co sensitization has been observed [102]. Reproductive toxicology has been noted at the neuroendocrine and gonadal levels, with Ni observed to substitute for other metals in metal dependent enzymes, leading to altered protein function [106]. Nickel can readily cross cell membranes via Ca channels and compete with Ca at its receptors, and can cross-link amino acids to DNA, leading to ROS [106]. Many pathogenic effects of Ni are due to the interference with the metabolism of essential metals such as Fe(II), Mn(II), Ca(II), Zn(II), and Mg(II) [101]. Male mice orally administered NiSO_4_ displayed lowered sperm count and motility, as well as altered testicular enzymes [106]. Nickel essentiality is related to a number of roles, including the formation of cyclic guanosine monophosphate, a signaling molecule that regulates various physiological processes such as blood pressure, sperm physiology, sodium metabolism, and cardiovascular health [107]. Additionally, Ni is present in RNA, and is bound to proteins such as insulin and keratin, amino acids, and serum albumin [107]. Nickel activates several enzymes, and Ni deficiency has been accompanied by histological and biochemical changes and reduced iron resorption leading to anemia, also influencing Zn metabolism [108]. Nickel deficiency in animals has been associated with growth reduction, anemia, reduced reproductive output, and reduced enzyme activity [103]. However, evidence for Ni’s essentiality in aquatic animals is circumstantial [103].

Nickel toxicity varies among fish species [103]. The toxicity typically ranks low and is equally toxic to Zn under acute conditions [103]. Several studies have demonstrated that Ni is much less toxic in hard water than in soft water, with the rationale that Ca and Mg protect fish generally by competing for binding sites, such as those on the gill [103]. During waterborne exposures, fish can take up Ni through the gills or olfactory epithelium or by the gut through diet [103]. It is transported throughout the fish by blood while bound to albumins and short peptides, and is found at low concentrations in all tissues; Ni preferentially accumulates in the kidneys with overexposure [103]. Acute Ni toxicity is associated with branchial lesions that cumulatively increase the diffusive distance across the gill epithelium, leading to impaired respiratory functions. Aquaporins are membrane-channel proteins selectively permeated by water and small solutes in organisms from prokaryotes to mammals; they may be up- or downregulated and are influenced by ions, with Ni and Zn influencing water permeability in *Xenopus* oocytes [103,109,110]. In fish, molecular structure data indicate aquaporins that display similar functional properties as in tetrapods, thus their functional properties are conserved [110]. However, cell localization and experimental studies imply that the physiological roles of piscine aquaporins extend at least to osmoregulation, reproduction, and early development [110]; specific functional studies are needed. In kidneys, Ni can cause lesions in the renal tubules and antagonizes Mg reabsorption, likely in part because both elements share uptake transporters [103]. Nickel contamination in freshwater ecosystems is poorly understood, but Ni has the capacity to be genotoxic to fish and can cause oxidative damage to cells [111]. Biomarkers of exposure may be the amount of Ni bound to or accumulated in gill tissue, as well as behavior such as locomotory function [103]. Deeper knowledge of the basic physiology of Ni in fish—both as a nutrient and a toxicant—is needed.

### 3.5. Zinc

Zinc is found in many environmental compartments. It occurs in the atmosphere from windborne dust, fires, and volcanic emissions [112]. It is also common in soil and water and is more soluble than Ni or Cu, which have similar crustal abundance [61]. Zinc naturally occurs mainly as Zn oxide or the mineral sphalerite (ZnS) [113]. It has only one primary oxidation state (Zn^2+^), and the pH of most freshwaters facilitates Zn adsorption onto particulates [112]; surface water concentrations are usually <10 µg/L, while in groundwater it is ~10–40 µg/L [61]. In soils, Zn concentrations are influenced by weathering [112], and form compounds such as Zn oxide, chloride, sulfide, sulfate, phosphate, and borate [114]. Unlike other transition elements, Zn is relatively stable in the divalent state and does not undergo redox changes [115]. Zn has a HBSL for drinking water of 5 mg/L and a sediment PEC of 459 mg/kg (dry weight).

Zinc has been used for millennia in brass (an alloy of Cu and Zn) and bronze, and by many industries [114]. It remains a component of the alloy to make U.S. one-cent coins, it is applied as a protective anti-rust coating for other metals, and in paint [113]. Zinc is released into the environment from mining, smelting ores, steel production, coal burning, and burning of wastes [113]. Zinc oxide nanoparticles have been considered non-toxic and are biocompatible and are widely used in cosmetics (e.g., sunscreen), rubber, medicine, chemical fibers, electronics, and other industries [116]. With novel strategies for energy storage and conversion, Zn’s use in metal–air batteries holds promise [117].

Zinc is essential to all cells in all known organisms, and it is the second most abundant trace element in most vertebrates after Fe, and is the most abundant transition metal in humans [118,119]. Zinc enters the human food chain directly through the consumption of plants and indirectly through the consumption of animal products, and by drinking water [113,120]. The human body uses Zn more diversely than Fe, and often in minute quantities [118]. Being redox neutral, Zn binds to proteins with the appropriate amino acid motifs (~3000, or 10% of all proteins) and there is a cytosolic pool for cell signaling [112,118]. It is involved in numerous biological functions and is considered a multipurpose trace element [121]. Zinc is essential for many enzymes (e.g., alcohol dehydrogenase, alkaline phosphatase, carbonic anhydrase, leucine aminopeptidase, and superoxide dismutase), proteins, and DNA and RNA metabolism. Deficiencies cause neurodevelopmental disorders, hormonal imbalance, dermatitis, impaired reproductive capacity, delayed development, retarded growth, dementia, and immune dysfunction [112,113,122]. Additionally, Zn has important roles in biochemical pathways and cellular functions, such as the response to oxidative stress, homeostasis, DNA replication, DNA repair, cell cycle progression, apoptosis and aging [121]. Zinc may enter the cell through a number of different Zn transporters, which include Znt5 and several members of the Zip family of proteins, with their distribution and activities determining the supply of Zn within cells and animals [118]. The absorption of Zn is an active process facilitated by Zn transporter ZIP4, where the gene expression of ZIP4 and other ZIP family genes increases when a Zn deficiency exists [112]. Human Zn deficiencies due to nutritional factors and the related disease states have been documented globally [115]. Phytate-rich foods (anti-nutritional factors, implicated with the impaired absorption of minerals due to chelation) can decrease the availability of Zn from omnivorous diets [112,115].

Zinc toxicity is primarily affiliated with habitual excessive consumption of Zn-containing dietary supplements [112]. Zinc, similar to other metals, induces the synthesis of MT, proteins involved with Zn homeostasis and protection against heavy metal toxicity and oxidative damage [119]. That they occur ubiquitously in bacteria, invertebrates, and vertebrates attests to the importance of this structural protein motif, with MTs being increasingly recognized as fulfilling diverse functions. In homeostasis, the MTs bind or release Zn, with MT gene expression being related to Zn accumulation in certain organs [123]. The MTs participate in an array of protective stress responses [124]. Metallothionein gene and protein expression can be increased after essential- and toxic metal exposure, which may differ per tissue. Expressed in most mammalian tissues, several MT isoforms of MT exist with their roles delineated by organ and cellular sensitivity to the metal and its toxicity [125]. Even an early phase of a common human viral infection has been found to increase MT in organs, and MT are involved with redistribution of both essential and non-essential trace elements [124], thus attesting to the ubiquity as well as the complexity of MT as a biomarker. Additionally, other protective compensatory molecular systems such as stress proteins and glutathione synthesis may alter the expected shape of a dose-response curve for metal exposure [126]. Excess dietary Zn causes secondary Cu deficiency by influencing its bioavailability, with single high doses causing vomiting and other gastrointestinal symptoms [112]. The effects of inhalation exposure to Zn and Zn compounds vary, with the majority of effects in the respiratory tract [113]. Toxicity associated with industry includes flu-like metal fume fever from inhalation of Zn oxide; highly toxic Zn chloride fumes can result from industry as well as from smoke bombs for crowd control [112].

Fish require Zn as a micronutrient and, as with humans, it can be obtained from water and especially the diet [61,127]. Fish are moderately sensitive to waterborne Zn, with acute toxic concentrations being higher than for metals such as Ag, Cd, and Cu, but lower than those for Mn and Ni [34]. This relatively high risk of toxicity to aquatic life has led to Zn’s inclusion as a priority pollutant by the U.S. EPA [12]. Zinc may enter cells through L-type or epithelial Ca channels, of which the latter is believed to be important in gill Zn uptake at lethally toxic water concentrations, with the kidney also a target [22,118]. Zinc uptake can interrupt Ca^2+^ uptake, leading to hypocalcemia and death [128]. A first sign of gill damage is detachment of chloride cells from underlying epithelium [22]. In a study of the uptake of metals, livers of different fish species showed higher concentrations than muscle, with variability in levels among species and locations. More detailed studies at the organ level are needed to relate water and fish consumption to human health risks [34]. In studies with adult zebrafish, *Danio rerio,* exposed to Zn oxide nanoparticles, the Zn ions, and not the particle size, were the dominant toxicity factor [129]. Concentrations of Zn varied per organ, and the extracellular Zn^2+^ passed through the cell membrane, entered the cytoplasm, and bound to mitochondria, inducing ROS production by interfering with the electron transport chain; this chain of events caused mitochondrial dysfunction and activates apoptotic processes [129]. In research focusing on extracts of spent batteries (containing element mixtures such as Ni, Pb, Cu, and Zn) and their potential effects on early life stage zebrafish, the survival, hatching rate and body length decreased, heart rate increased, and heat shock protein (HSP70) and MT gene expressions were inhibited, thus decreasing the ability to moderate stress [130]. In another organismal-level biomarker study, the anadromous alewife, *Alosa pseudoharengus,* from the Gulf of Maine, experienced growth reduction and spine curvatures as well as heavy metal accumulation in organs, with Zn in liver as high as 38.0 μg/g, indicating heavy metal pollution in rivers and surrounding marine waters [131]. In a study using a plant material (i.e., date seeds) to adsorb waterborne Zn, various antioxidant biomarkers were effective in assessing exposures in common carp, *Cyprinus carpio,* over 8 weeks [132]. Other treatments for the protection of fish and aquatic systems from heavy metals include activated carbon, ozonation, ultraviolet light, and reverse osmosis. More research could help advance understanding the balance of water-borne and diet-borne Zn contributions and for understanding bioavailability and toxicity for various fish species under various environmental conditions [127].

## 4. Rare Earth Elements

Rare earth elements (REEs, or lanthanides) are widely distributed in nature in the form of minerals and are non-essential for life with no known biological functions (Figure 1) [133]. Scandium (Sc) and yttrium (Y), which are transition metals, have a similar physiochemistry to the lanthanides and are commonly found in the same mineral assemblages, and so are often considered REEs [134,135,136]. Due to their analogous properties, the REEs tend to occur together in ores, along with traces of uranium and thorium [135,137]. Separation of REEs from each other during processing and refining is challenging, as they rarely occur in concentrated forms, thus presenting financial and technical challenges related to their acquisition for use [7,134]. Contrary to their name, REEs are not rare, with the appellation referring to their lack of purity in ore deposits [134,138]. They are divided into light and heavy REEs according to their atomic number [133]. One of the most important properties in determining their chemical behavior is cation size; the size of lanthanide cations decreases with increasing atomic number [7].

The REEs are soft, silver-colored metals with high melting points [7]. Uses include metallurgical applications and alloys, electronics, chemical catalysts, phosphors, automotive catalytic converters, permanent magnets, petroleum refining catalysts, and feed additives and fertilizers in China [134,139]. Rare earth element nanoparticles play roles in cosmetic- and consumer products, polishing materials and automobile exhaust catalysts [140]. In nature, REEs almost always occur in the +3 valency state [7], though additional oxidation states of cerium (Ce) and europium (Eu) (Ce^4+^ and Eu^2+^) are not uncommon.

The REEs are central in critical technologies, thus their use and extraction are increasing, as is their release into agricultural, aquatic and soil ecosystems to potentially reach and expose humans [133,141]. Understanding the fate, bioavailability, and toxicity of these compounds has recently gained increased attention. The ionic radii of lanthanides are similar to Ca^2+^, Y^3+^, Cu, Zn, and Fe, depending on ionization state. Hence, the REEs may be capable of interacting with both Ca and heavy metal binding sites. Thus, some REEs could compete with Ca binding sites, altering cell functions; for example, lanthanum (La) is a Ca channel blocker and may disturb the mitochondrial membrane potential and electrolyte gradients [141]. Conversely, the close ionic radius to Cu, Zn, and Fe confers reactivity towards electron rich compounds such as thiol, nitrogen, and oxygen. Thus, REEs could deplete cysteine or glutathione pools needed for redox maintenance and synthesis of MT [141]. In the clinical arena, a human health treatment developed with a REE (erbium (Er) laser) has been shown to improve vaginal health and sexual function in postmenopausal women, and Y-90 decay provides unique delivery in a radiation therapy for hepatocellular carcinoma [142].

Toxicology studies for REEs, employing “early warning” biomarker responses, have revealed dose-response curves that deviate from linearity, and are unpredictable by classical toxicology [143,144]. For example, endocrine disrupters, a large group of synthetic chemicals able to interact with cellular hormone receptors, at times show non-monotonic dose-response curves, meaning the slopes change from negative to positive or vice versa, where lower (or higher) doses can pose increased risk [143,144,145]. Hormesis is the phenomenon in which an agent produces harmful biological effects at moderate to high doses, but has beneficial effects at low doses [143]. The REEs may appear to display biphasic redox behavior, where at low concentrations/exposure times they act as anti-oxidants, which disappear and produce oxidative stress at high concentrations/longer exposure times [141], potentially following hormetic concentration-related trends. This may suggest that REEs have stimulatory or protective effects at low levels, then induce adverse effects at higher concentrations [143,146].

Despite REEs widespread applications, few ecotoxicology studies have been performed [136,138,147]. No REEs are listed as toxic substances in the Toxic Substances and Disease Registry (https://www.atsdr.cdc.gov/) [accessed on 8 August 2022]. Limited studies on human health effects have been done, with most conducted with Ce and La [146,147]. One study documented blood parameter changes after REE exposure via food [148]. Mechanisms of REE-associated health effects relate to modulating oxidative stress [146]. Adverse outcomes include endpoints such as growth inhibition, cytogenetic effects, and organ-specific toxicity [146]. Blood biochemical profiles from people having high environmental REE exposure showed low total serum protein, albumin beta-globulin, glutamic pyruvic transaminase, and triglycerides alterations in immunoglobulin concentrations, and high cholesterol and arteriosclerosis. Males were able to reverse some adverse blood responses [148]. In selected *in vitro* toxicological endpoints with tests of REEs, no significant potential of mutagenicity, skin irritation and sensitization or endocrine disruption were noted for humans; with results from a *Tubifex tubifex* worm showing movement inhibition similar to those induced by Cd, the authors concluded that REEs likely exert acute and chronic toxic effects in the aquatic environment [149].

Metal oxide nanoparticles (MONPs), some formulated with REEs, are valuable in many industrial sectors, especially healthcare, resulting in increases in human exposures [150]. Several MONPs, including Ce oxide (CeO_2_), have antibacterial properties [150]. Rare earth element-containing MONPs can cross the blood-testis barrier, accrue in the testis [150,151] and interrupt spermatogenesis, a vulnerable process occurring in a protected environment. Mutations can occur in germ cells, thus influencing fertilization, embryogenesis, or even biological effects in offspring [150]. Direct effects of CeO_2_ NP on human and mouse spermatozoa have been observed [152,153]. The gas-phase processes used for manufacturing commercial quantities of a wide range of sub-micron sized nanoparticles can expose people to aerosolized particles and aggregates [138]. Cerium oxide nanoparticles have been found to penetrate cellular membranes, with 80% of total CeO_2_ being detected intracellularly within 24 h incubation in alveolar cell culture [151]. Some CeO_2_ nanoparticles developed have antioxidant properties, and thus are used pharmacologically for treatment of diseases associated with oxidative stress [140]. With Ce being redox active, the CeO_2_ nanoparticles can induce changes in intracellular redox status, leading to cancer cell death, while causing little inflammation [140]. In an inhalation study with nano-sized neodymium (Nd) oxide in male rats, the target organ was the lungs, with several histopathological and blood and serum parameters. These included altered neutrophil and macrophage numbers, being related to dose and exposure duration [154]. Information describing human adverse health effects derived from epidemiology studies is still severely lacking, with realistic exposure levels for nanoparticles needed [140].

Studies in aquatic systems in which significant waste releases might occur are particularly relevant [134]. In arid ecosystems, reclamation and reuse of municipal wastewater are important components of the water budget [120]. Applied to waters to manage phosphorous, geo-materials based on aluminum (Al) or La are applied, with human health effects due to Al much more well studied than La [155]. For non-target aquatic organisms, studies surrounding REE toxicity have increased since ~2020, yet there is still a scarcity of information, even for the more commonly used samarium (Sm) and Y [138]. The REEs exhibit lower bioaccumulation in fish relative to aquatic plants, with concentrations higher in internal organs and skeletons than in muscles [120,136]. Rare earth elements (La, Ce, Sm, gadolinium (Gd), Y) are adsorbed rapidly to sediments, with bioaccumulation in biota found to be duckweed > crustacean > shellfish > fish [156]. In livers of ytterbium (Yb)-exposed goldfish, *Carassius auratus*, similar to other hormetic effects noted with REEs, glutamate-pyruvate transaminase was stimulated at 0.05 mg/L Yb^3+^ and inhibited at higher concentrations [157]. Other liver enzyme endpoints yielded differential responses with exposure time, with catalases showing promise as a sensitive biomarker of exposure [157]. In a case study of juvenile rainbow trout exposed to REEs, changes were detected at the gene expression level and compared with adverse effects at the organ or organismal level [143]. After exposure to selected REEs for 96 h at 0.7 mg/L, mortality was Y < Sm < Gd = Er < Nd = Ce [143]. All the REEs induced stress response genes for HSP70, PCNA, GADD45 and SOD, while expressions of MT, CYP1A1 and GST were influenced by 5 out of 7 REEs [143]. This study suggested that exposures of low concentrations of REEs to rainbow trout lead to changes in protein conformation, cell proliferation, and DNA repair, and the identified AOP were informed with gene expression results and the acute toxicities [143].

Overall, these studies exemplify the need for close study of the REEs, as they display variable modes of action and non-linear toxicological mechanisms, whereby controlled studies can help to delineate toxicity, supporting safe handling as well as informing ecosystem management actions. Gene targets involving oxidative state, genotoxicity, divalent cation binding, hematological, MT and other protein responses are notable targets for further study.

## 5. Discussion

Mineral commodities have been essential ingredients for building and enhancing civilizations from the Stone Age to the present [5]. While the biogeochemical cycles of some elements such as carbon, mercury, and Pb have been well characterized, the cycling of most of the CMs is poorly understood. With the increased use and demand for CMs, a quantitative understanding at the landscape level of the flow of materials dominated by human action can support the understanding of the long-term availability of resources, and the potential for toxicity to ecosystems [158]. This review lends to a clearer understanding of the global cycles of several CMs by presenting information on their occurrence, use, exposure and toxic effects in humans and fish. This is only one step in understanding a segment of the flow of elements as a part of the CM global cycles.

Efforts to ensure secure and reliable supply of CMs necessitates understanding their potential health and ecosystem effects. Even with benchmark guidelines, regulations, laws, and policies in place for environmental stewardship and human health protection for some CMs, increased contact with CMs and CM occurrence in aquatic ecosystems are assured. Though complicated by the specifics of metal bioavailability and bioreactivity that are influenced by a multitude of ecological conditions influencing water chemistry, the concept that biological activity of a compound is a direct function of its chemical structure is well founded [159]. Nevertheless, delineating an AOP can be less than straightforward for the CMs under variable conditions such as ambient water quality, yet they may be postulated. Predicting potential health outcomes for humans and fish could be advanced with a better understanding of factors that influence speciation and bioavailability and incorporating some constructs of the biotic ligand model (BLM) to better understand metal interactions in site-specific environments—such as predictions of the degree of metal binding at the site of action—being in turn related to a toxicological response [160]. The BLM has contributed to the understanding of physiological mechanisms for acute toxicities, assisting in setting water quality standards [55]. The information presented in this review may be helpful in predicting potential health outcomes of CM exposures.

Critical Minerals that are essential elements (Co, Cr, Mn, Ni, and Zn) tend to have many published scientific resources available compared to the REEs. This is likely because essential elements are needed for life. Compared with the essential elements, REEs have been the subject of fewer toxicology-oriented studies, occurring primarily after 2010, with most focusing on Ce and La.

Biomarkers are signaling events in biological systems, with ‘biomarkers of exposure’ being either an exogenous substance within a system, or the interactive product between a xenobiotic compound and endogenous components; ‘biomarkers of effect’ are endogenous components of the animal, a measure of its functional capacity, or an altered state of that system that is recognized as impairment or disease [21]. These quantitative measures are useful together with tissue burdens of contaminants [161]. Understanding events along an AOP following exposure is foundational to determining CM-associated risks and can provide insight into relevant research avenues and CM exposure risks.

Biomarkers of exposure for CMs include metals found in biological tissues [41]. An adequate intake of essential trace elements via food is required in order to avoid deficiency states, though excess and toxic exposure can occur by a variety of mechanisms, including food intake and inhalation in humans, and through gill and gut in fish [122]. Biomarkers of exposure for trace elements include metals in biological matrices such as toenails, bone, organs, hair, bodily fluids, fish scales, and bird feathers, as well as proteins such as metallothionein. Biomarkers of effect often include respiratory symptoms, neurotoxicity, genotoxicity, apoptosis, and mitochondrial dysfunction in humans, and gill irritation, decreased growth (in fish), altered blood parameters, and reproductive toxicity.

Of the CMs considered in this review, all of the essential nutrients and most (56%) of the REEs are included on the Agency for Toxic Substances and Disease Registry Substance Priority List (Table A2) [14]. Substances on this list are of great human health concern. Of the CMs considered, four of the essential nutrient elements are included in compounds in the 2021 list in the Report on Carcinogens [13]. That the CMs are on these lists underscores the need for more research, especially as this topic is in accord with One Health, a transdisciplinary approach that considers the relationship between humans, animals, and the environment, with a special link being water [162].

Metal ions are unique among nutrients because they cannot be synthesized or degraded by metabolism [19]. Many enzymes require metals for catalytic activity [15,16], and although essential, they can be toxic and pose serious health risks if not processed in the body properly, or if they bioaccumulate at sufficiently high concentrations [15,17,18]. For animal physiology and toxicology, metal speciation is highly important, influencing cellular interactions and entry into cells [19,42].

The occurrence of adverse health effects from essential trace elements depends on such things as dose, exposure conditions, bioavailability, and chemical species, with toxicity induced if the exposure is excessive or if the chemical species or exposure route is other than physiologically needed [122]. In both general and occupational or “high concentration” environments, exposure situations are complex, resulting in potential enhancement or suppression of an adverse effect by interactions among metals, water quality parameters (for fish, especially), and organic compounds. Nonetheless, in spite of these complexities, we observe some broad generalizations:

- At the cell surface, the critical first points of contact are lipids and biomembranes, with influences on bilipid membrane morphology and asymmetry.
- For the essential nutrients, the initial events that can be linked to an AOP can be considered to be potentially conserved across animal taxa [55].
- In fish, acute exposures target gills, which generally comprise over 50% of the surface area of the animal and are in intimate and continuous contact with the external water [19].
- The transport mechanisms into cells are varied. Metal-specific protein ion channels are needed for influx and efflux of Co, Mn, and Ni, and some others mediate their transport as well as allow influx of other divalent cations [42]. To excrete excess metals, various efflux mechanisms are upregulated so as to release them to the extracellular matrix [42]. Non-specific phosphate/sulfate anion transporters are involved in Cr(VI) transport [72,78], and divalent metal transporters (DMT) can transport Zn^2+^, Mn^2+^, Co^2+^, Cd^2+^, Cu^2+^, Ni^2+^, Pb^2+^, and Fe^2+^ [85]. Nickel can readily cross cell membranes via Ca channels and compete with Ca at its receptors [106]. The absorption of Zn is an active process facilitated by Zn transporters (ZIP4) [112].
- Cr(III) cannot cross cell membranes; Cr(VI), which can cross membranes, is more toxic to humans and is a carcinogen.
- In fish, compared with humans the molecular mechanisms of metal toxicity and subsequent physiological effects are under-studied [57]. Moreover, controlled studies with fish and CM can delineate molecular pathways and so too inform human AOP.
- Studies thus far imply REEs have stimulatory or protective effects at low levels, then induce adverse effects at higher concentrations [143,146].
- By virtue of their ionic radii, the REEs may be capable of interacting with binding sites for Ca and heavy metals, disrupting normal protein function, or may deplete antioxidant protein pools [141].
- Despite REEs widespread applications, few ecotoxicology studies have been performed [136,138,147].
- Because the REEs display variable modes of action, can be competitive with other metals at the cellular level, and display non-linear toxicological mechanisms, more studies with fish and humans can help to delineate AOP.

## 6. Summary

This review summarizes potential AOPs for human and fish receptors for essential trace metals and REEs that are also considered to be CMs. The combined use of biomarkers of effects and biomarkers of exposure contributes to an improved understanding of environmental impacts of increased production and use of these CMs. This is the first attempt to consider CMs from a viewpoint that anticipates and attempts to predict AOP, as increased exposures will likely occur with increased use. Factors that may complicate interpretation of biomarkers of effect include chemical species differences, as well as human and ecological differences in parameters of nutritional status, genetic susceptibility, and compensatory molecular systems of response. Nevertheless, the use of biomarkers, along with biomonitoring —whereby information is obtained on exposure from all sources and via all absorption routes —can be an effective tool in risk management of CM exposures for humans and for fish.

## Figures and Tables

**Table 1:** The U.S. Geological Survey 2022 list of critical minerals ^1^.

Aluminum (Al)	Graphite (C)	Rubidium (Rb)
Antimony (Sb)	Hafnium (Hf)	Ruthenium (Ru)
Arsenic (As)	*Holmium (Ho)*	*Samarium (Sm)*
Barite (barium; Ba)	Indium (In)	*Scandium (Sc)*
Beryllium (Be)	Iridium (Ir)	Tantalum (Ta)
Bismuth (Bi)	*Lanathanum (La)*	Tellurium (Te)
*Cerium (Ce)* ^2^	Lithium (Li)	*Terbium (Tb)*
Cesium (Cs)	*Lutetium (Lu)*	*Thulium (Tm)*
* Chromium (Cr)	Magnesium (Mg)	Tin (Sn)
* Cobalt (Co)	* Manganese (Mn)	Titanium (Ti)
*Dysprosium (Dy)*	*Neodymium (Nd)*	Tungsten (W)
*Erbium (Er)*	Nickel (Ni)	Vanadium (V)
*Europium (Eu)*	Niobium (Nb)	*Ytterbium (Yb)*
Fluorspar (Fluorine; F)	Palladium (Pd)	*Yttrium (Y)*
*Gadolinium (Gd)*	Platinum (Pt)	* Zinc (Zn)
Gallium (Ga)	*Praseodymium (Pr)*	Zirconium (Zr)
Germanium (Ge)	Rhodium (Rh)	

^1^ 2022 Final List of Critical Minerals. 87 Fed. Reg. 10381. 24 February 2022. ^2^ Rare earth elements are italicized. * Nutritionally essential trace elements per considerations this manuscript.

## Data Availability

No new data were created or analyzed in this study. Data sharing is not applicable to this article.

## Appendix A

**Table A1:** Critical Element forms listed in the 15th Report on Carcinogens, 2021 ^a^.

CAS ^b^	Substance Name	Listing ^c^
1309-64-4	Antimony Trioxide	RAHC
7440-38-2	Arsenic	Known
7440-41-7	Beryllium	Known
7787-47-5	Beryllium Chloride	Known
7787-56-6	Beryllium Sulfate Tetrahydrate	Known
13327-32-7	Beryllium Hydroxide	Known
13510-49-1	Beryllium Sulfate	Known
13598-00-0	Beryllium Silicate	Known
13598-15-7	Beryllium Phosphate	Known
25638-88-4	Zinc Beryllium Silicate	Known
66104-24-3	Beryllium Carbonate	Known
1333-82-0	Chromium Trioxide	Known
7775-11-3	Sodium Chromate	Known
7778-50-9	Potassium Dichromate	Known
7788-98-9	Ammonium Chromate	Known
7789-00-6	Potassium Chromate	Known
7789-06-2	Strontium Chromate	Known
7789-09-5	Ammonium Dichromate	Known
10588-01-9	Sodium Dichromate	Known
11119-70-3	Lead Chromate	Known
13530-65-9	Zinc Chromate	Known
13765-19-0	Calcium Chromate	Known
18540-29-9	Chromium (VI)	Known
71-48-7	Cobalt Acetate	RAHC
7440-48-4	Cobalt	RAHC
7646-79-9	Cobalt Chloride	RAHC
10026-24-1	Cobalt Sulfate Heptahydrate	RAHC
10124-43-3	Cobalt Sulfate	RAHC
10141-05-6	Cobalt Nitrate	RAHC
11104-61-3	Cobalt Oxide	RAHC
12653-56-4	Cobalt Sulfide	RAHC
NA	Cobalt-Tungsten Carbide: Powders and Hard Metals	RAHC
373-02-4	Nickel Acetate	Known
1313-99-1	Nickel Monoxide	Known
7440-02-0	Metallic Nickel	RAHC
7786-81-4	Nickel Sulfate	Known
11113-75-0	Nickel Sulfide	Known
12035-72-2	Nickel Subsulfide	Known
12054-48-7	Nickel Hydroxide	Known
NA	Nickel Compounds	Known

^a^ The Report is a scientific and public health document that identifies and discusses agents or exposure circumstances that may pose a cancer hazard to humans. (https://ntp.niehs.nih.gov/go/roc15; accessed on 5 June 2022); ^b^ Chemical Abstracts Service registry number. ^c^ RAHC = Resonably anticipated to be a human carcinogen; Known = Known to be a human carcinogen.

**Table A2:** Critical Element forms listed on the 2019 Agency for Toxic. Substances and Disease Registry Substance Priority List ^a^.

Ranking	Substance Name	CAS ^b^
1	Arsenic	7440-38-2
14	Chromium (VI) hexavalent	18540-29-9
43	Beryllium	7440-41-7
58	Chromium (VI) trioxide	1333-82-0
70	Cobalt	7440-48-4
94	Nickel	7440-02-0
136	Barium	7440-39-3
150	Chromium	7440-47-3
155	Fluorine	7782-41-4
164	Zinc	7440-66-6
173	Palladium^c^	7440-05-3
188	Manganese	7439-96-5
228	Cesium-137	10045-97-3
244	Antimony	7440-36-0
277	Vanadium	7440-62-2
281	Aluminum	7429-90-5
351	Titanium	7440-32-6
359	Lithium	7439-93-2
360	Chromium, trivalent	16065-83-1
362	Tin	7440-31-5
376	Cesium-134	13967-70-9
384	Tungsten	7440-33-7
448	Indium^c^	7440-74-6
470	Cerium	7440-45-1
476	Neodymium	7440-00-8
479	Germanium^c^	7440-56-4
492	Bismuth-214	14733-03-0
494	Bismuth-212	14913-49-6
510	Ytterbium	7440-64-4
511	Scandium	7440-20-2
513	Gallium	7440-55-3
514	Niobium	7440-03-1
519	Tellurium	13494-80-9
525	Cobalt-60	10198-40-0
545	Chromium (IV)	15723-28-1
550	Europium	7440-53-1
551	Dysprosium	7429-91-6
551	Praseodymium	7440-10-0
551	Samarium	7440-19-9
566	Aluminum oxide	1344-28-1
578	Cesium	7440-46-2
578	Lanthanum	7439-91-0
578	Magnesium	7439-95-4
578	Rubidium	7440-17-7
578	Yttrium	7440-65-5
578	Zirconium	7440-67-7
692	Tantalum ^c^	7440-25-7

^a^ The list includes substances determined to be of greatest public health concerns to persons at or near national priority list sites. (https://www.atsdr.cdc.gov/spl/index.html#2019spl; accessed on 30 May 22). ^b^ Chemical Abstracts Service registry number. ^c^ Included on version 1 of the 2019 list.
